# Supplementary material for: Dynalign II: common secondary structure prediction for RNA homologs with domain insertions
Source: Nucleic Acids Res. 2014 Nov 21;42(22):13939–48. doi: 10.1093/nar/gku1172 (PMC4267632; doi:10.1093/nar/gku1172)
Supplement: SUPPLEMENTARY DATA [file supp_gku1172_nar-02021-z-2014-File012.zip › manual/Overview/Object_Files.html]

RNAstructure Installation and Overview: Object Files


|  |  |  |
| --- | --- | --- |
|  | RNAstructure Installation and Overview Object Files | - Contents - Index |
| A special note on object files  When building the RNAstructure text interfaces and then the RNAstructure GUI back to back, incorrect object files may be incorporated in the second interface that is built.  That is, if the RNAstructure text interfaces are built first, an immediate build of the RNAstructure GUI may incorporate incorrect object files, and if the RNAstructure GUI is built first, an immediate build of the text interfaces may incorporate incorrect object files.  To eliminate any possible build or runtime errors stemming from this, use the Makefile in the RNAstructure/ directory and execute the command "make clean" after the first build is complete, so resources are made anew for the second build. | | |
| Visit The Mathews Lab RNAstructure Page for updates and latest information. | | |
